# Supplementary material for: Metabolomics window into the role of acute kidney injury after coronary artery bypass grafting in diabetic nephropathy progression
Source: PeerJ. 2020 May 14;8:e9111. doi: 10.7717/peerj.9111 (PMC7231503; doi:10.7717/peerj.9111)
Supplement: Table S1 — Note: Metabolites between two groups were considered different when the p-value of the T test < 0.05 and VIP ≥ 1. Abbreviations: CABG, Coronary artery bypass grafting; A, preoperative sample; B, postoperative sample; VIP, variable importance in projection. [file peerj-08-9111-s001.docx]

Supplementary Table. Differential metabolites of DM patients after CABG procedure.

| metabolite | Class | ID | Ion mode | Iog2 fold change (B mean / A mean) | Change trend of B vs A | P value | VIP |
| --- | --- | --- | --- | --- | --- | --- | --- |
| Pantothenol | Coenzymes and vitamins | MEDP249 | Positive | 22.554 | up | 0.016 | 2.695 |
| 5-iso PGF2VI | Oxidized lipid | MEDN780 | Negative | 12.398 | up | 0.000 | 2.370 |
| Trans-4-hydroxy-L-proline | Amino Acid | MEDP083 | Positive | 6.426 | up | 0.001 | 1.991 |
| Catechol | Phenols | MEDP892 | Positive | 5.304 | up | 0.027 | 1.220 |
| Dulcitol | Carbohydrate | MEDN120 | Negative | 5.258 | up | 0.005 | 1.722 |
| α-Ketoglutaric Acid | Amino Acid | MEDN202 | Negative | 5.175 | up | 0.017 | 3.992 |
| D-Sorbitol | Carbohydrate | MEDN213 | Negative | 5.129 | up | 0.004 | 1.424 |
| Uridine triphosphate (UTP) | Nucleotide | MEDN609 | Negative | 5.118 | up | 0.020 | 1.829 |
| L-Alanyl-L-Lysine | Amino Acid | MEDP087 | Positive | 4.880 | up | 0.004 | 1.199 |
| L-Carnitine | Camitine | MEDP205 | Positive | 4.854 | up | 0.003 | 4.534 |
| Riboflavin | Coenzymes and vitamins | MEDP250 | Positive | 4.769 | up | 0.013 | 3.232 |
| DL-Carnitine | Camitine | MEDP523 | Positive | 3.861 | up | 0.001 | 6.265 |
| 3-Methylglutaric acid | Organic Acid | MEDN544 | Negative | 3.401 | up | 0.024 | 3.838 |
| 2,2-Dimethyl Succinic acid | Lipids_Fatty Acids | MEDN613 | Negative | 3.346 | up | 0.024 | 3.818 |
| 2-Methylglutaric Acid | Organic Acid | MEDN426 | Negative | 3.332 | up | 0.024 | 3.865 |
| 4-Guanidinobutyric Acid | Organic Acid | MEDP296 | Positive | 3.292 | up | 0.005 | 2.814 |
| Acetyl-L-carnitine | Camitine | MEDP510 | Positive | 3.040 | up | 0.000 | 9.247 |
| Phenylpyruvate | Benzene | MEDN862 | Negative | 2.376 | up | 0.002 | 1.023 |
| 2-Methylbutyroylcarnitine | Lipids_Fatty Acids | MEDP618 | Positive | 2.163 | up | 0.006 | 7.072 |
| P-Aminobenzoate | Benzene | MEDP450 | Positive | 2.160 | up | 0.042 | 1.983 |
| 2'-Deoxycytidine 5'-Diphosphate | Nucleotide | MEDP888 | Positive | 1.358 | up | 0.003 | 1.076 |
| Caffeic acid | Organic Acid | MEDN301 | Negative | 0.967 | up | 0.010 | 1.050 |
| Adrenochrome | Indole | MEDN672 | Negative | 0.791 | up | 0.014 | 2.625 |
| Isobutyryl carnitine | Camitine | MEDP577 | Positive | 0.663 | up | 0.014 | 4.531 |
| Hippuric Acid | Organic Acid | MEDP315 | Positive | 0.181 | up | 0.048 | 1.668 |
| 2-(Dimethylamino)Guanosine | Nucleotide | MEDP380 | Positive | -0.299 | down | 0.043 | 1.932 |
| Uric Acid | Organic Acid | MEDP333 | Positive | -0.369 | down | 0.001 | 2.056 |
| Pipecolinic Acid | Organic Acid | MEDP358 | Positive | -0.527 | down | 0.044 | 1.450 |
| L-Dihydroorotic Acid | Organic Acid | MEDP321 | Positive | -0.587 | down | 0.033 | 1.653 |
| 3-Methylthiophene | Heterocyclic compound | MEDP726 | Positive | -0.616 | down | 0.024 | 2.202 |
| MESITYL OXIDE | Ketones | MEDP800 | Positive | -0.619 | down | 0.030 | 1.981 |
| Ethyl heptanate | Fatty acyls | MEDP657 | Positive | -0.627 | down | 0.024 | 1.505 |
| Isoamyl butyrate | Fatty acyls | MEDP656 | Positive | -0.636 | down | 0.020 | 1.569 |
| Cyclohexanone | Ketones | MEDP673 | Positive | -0.638 | down | 0.025 | 2.091 |
| Citric Acid | Amino Acid | MEDN198 | Negative | -0.654 | down | 0.046 | 2.539 |
| δ-Valerolactam | Pyridine | MEDP541 | Positive | -0.745 | down | 0.000 | 2.820 |
| Β-Pseudouridine | Nucleotide | MEDN434 | Negative | -0.811 | down | 0.022 | 1.503 |
| Tyrosine | Amino Acid | MEDP009 | Positive | -0.888 | down | 0.004 | 2.098 |
| Α-D-Glucose | Carbohydrate | MEDP223 | Positive | -0.911 | down | 0.013 | 1.494 |
| DL-O-Tyrosine | Amino Acid | MEDP508 | Positive | -0.941 | down | 0.012 | 1.342 |
| 2-Hydroxycinnamic acid | Benzene | MEDP507 | Positive | -1.022 | down | 0.009 | 1.255 |
| 2-(3,4-dimethoxyphenyl) ethanamine | Benzene | MEDP606 | Positive | -1.036 | down | 0.007 | 1.349 |
| Choline chloride | Coenzymes and vitamins | MEDP853 | Positive | -1.105 | down | 0.006 | 1.935 |
| Estrone | Hormones | MEDN524 | Negative | -1.180 | down | 0.002 | 2.952 |
| L-Norleucine | Amino Acid | MEDP519 | Positive | -1.331 | down | 0.002 | 3.745 |
| Phosphoric Acid | Organic Acid | MEDP423 | Positive | -1.453 | down | 0.007 | 1.296 |
| 3-Hydroxyhippuric Acid | Organic Acid | MEDP379 | Positive | -1.464 | down | 0.000 | 5.755 |
| 5'-Deoxy-5'-(Methylthio) Adenosine | Nucleotide | MEDP401 | Positive | -1.520 | down | 0.017 | 1.058 |
| Oxindole | Indole | MEDP546 | Positive | -1.569 | down | 0.037 | 1.228 |
| 4-Hydroxyhippurate | Benzene | MEDN816 | Negative | -1.584 | down | 0.005 | 2.163 |
| Salicyluric acid | Benzene | MEDN623 | Negative | -1.612 | down | 0.004 | 1.945 |
| -z x | Organic Acid | MEDN621 | Negative | -1.968 | down | 0.000 | 9.792 |
| 3-(3-Hydroxyphenyl)-3-hydroxypropanoic acid | Organic Acid | MEDN826 | Negative | -1.993 | down | 0.005 | 2.281 |
| Trigonelline | Coenzymes and vitamins | MEDP251 | Positive | -2.014 | down | 0.008 | 1.253 |
| 1-Aminopropan-2-ol | Alcohol | MEDP831 | Positive | -2.229 | down | 0.000 | 2.536 |
| Trimethylamine N-Oxide | Amino Acid | MEDP084 | Positive | -2.368 | down | 0.000 | 2.844 |
| 3-(2-Hydroxyphenyl) propionic acid | Organic Acid | MEDP562 | Positive | -2.376 | down | 0.000 | 1.358 |
| 3-(4-Hydroxyphenyl)-Propionic Acid | Benzene | MEDP111 | Positive | -2.406 | down | 0.000 | 1.406 |
| Hydroquinone | Phenols | MEDN647 | Negative | -3.002 | down | 0.002 | 1.370 |
| Azelaic acid | Organic Acid | MEDN300 | Negative | -3.338 | down | 0.000 | 2.768 |
| MARMESIN | Carbohydrate | MEDN560 | Negative | -4.088 | down | 0.035 | 1.145 |

Note: Metabolites between two groups were considered different when the p-value of the T test < 0.05 and VIP ≥ 1.

Abbreviations: CABG, Coronary artery bypass grafting; A, preoperative sample; B, postoperative sample; VIP, variable importance in projection.
