# Supplementary figures and images for: Metabolomics window into the role of acute kidney injury after coronary artery bypass grafting in diabetic nephropathy progression
Source: PeerJ. 2020 May 14;8:e9111. doi: 10.7717/peerj.9111 (PMC7231503; doi:10.7717/peerj.9111)

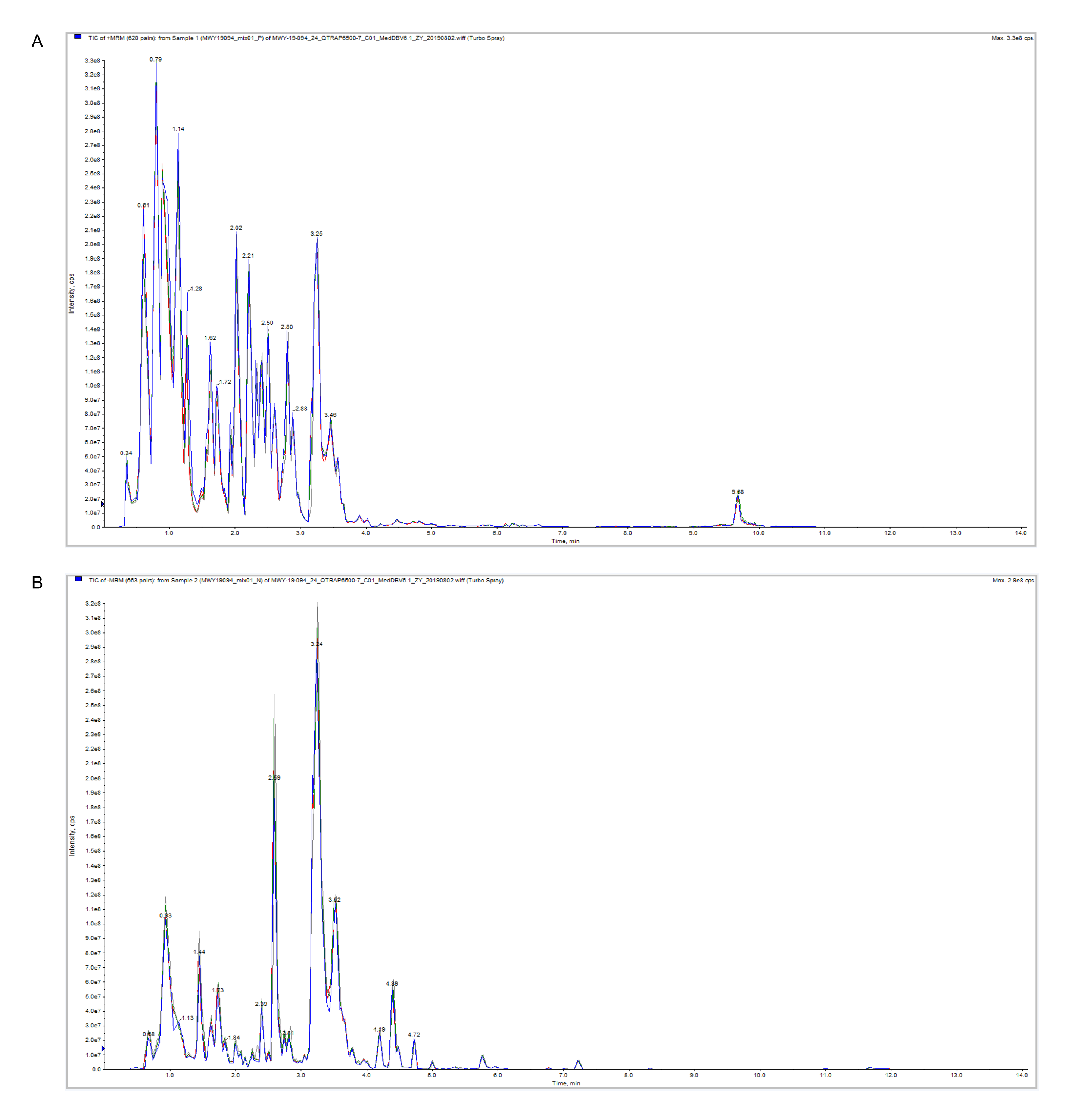

Supplement: Figure S1 — Positive ionization mode (A) and negative ionization mode (B) of the typical TIC from the QC samples. Abbreviation: TIC, total ion chromatograms; QC, Quality Control. [file peerj-08-9111-s002.png]

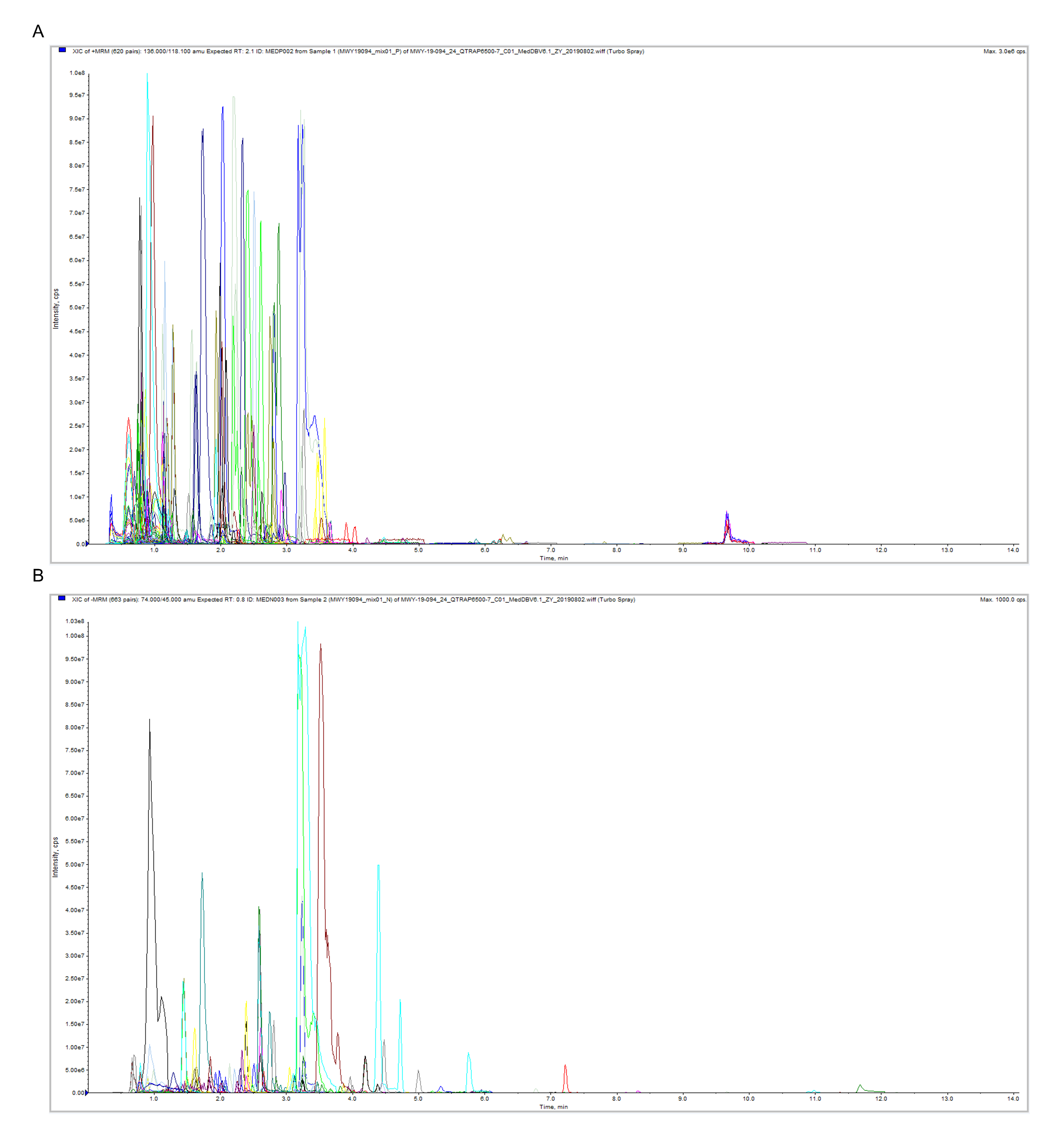

Supplement: Figure S2 — MRM spectra obtained from the QC samples with positive (A) and negative mode (B). Abbreviation: MRM, multiple reaction monitoring; QC, Quality Control. [file peerj-08-9111-s003.png]
